# Health Care Equity and *BRCA1/2* Testing

**DOI:** 10.1089/heq.2024.0167

**Published:** 2025-02-21

**Authors:** Steven Sorscher

**Affiliations:** Winston-Salem, North Carolina, USA.

**Keywords:** oncology, *BRCA1/2* testing, health equity

## Abstract

Although most cancers are sporadic, a significant proportion are related to inherited cancer-causing genes called pathogenic germline variants (PGVs). There are recommended measures for prevention and earlier diagnosis of cancers in patients identified as *BRCA1* and *BRCA2* PGV carriers, which are the most common cancer-predisposing PGVs. For example, published guidelines recommend that patients with *BRCA1/2* PGVs undergo bilateral oophorectomies to prevent ovarian cancer and regular magnetic resonance imaging to screen for breast cancer. Also, those same measures are recommended for family members identified by cascade testing as *BRCA1/2* carriers. Here, reports of the significant disparities between groups in which patients diagnosed with breast cancer are offered and undergo testing for *BRCA1/2* PGVs are reviewed. Expanding the current standard of care guidelines for *BRCA1/2* testing to all patients diagnosed with breast cancer and enacting the Cancer Moonshot 2.0 Initiative measures that should mitigate these disparities are discussed as well.

## Essay 

Roughly 5–10% of breast cancers are related to inherited cancer-causing genes called *BRCA1/2* pathogenic germline variants (PGVs). Patients diagnosed with breast cancer and their affected family members (FMs) who are also found to carry *BRCA1/2* PGVs are eligible for measures proven to prevent and diagnose earlier breast cancer (including second breast cancers) and other *BRCA1/2*-associated cancers.^1^

Major medical organizations have emphasized the need for equity in which patients are offered genetic testing for PGVs. The American Medical Association (AMA), American Cancer Society (ACS), the American Society of Clinical Oncology (ASCO), and the European Society of Medical Oncology (ESMO) all consider health equity to include equal opportunities for prevention and early detection of cancer.^2–5^ In 2020, ESMO President Solange Peters wrote that ESMO’s mission of health care equity includes “denouncing” all aspects of inequality and supporting access for cancer services to everyone regardless of “whether they can afford it, or their ethnicity or any other personal circumstance.”^5^

It would seem that health equity therefore relies on an equal opportunity for everyone who wishes to not only be evaluated for testing but then be tested for *BRCA1/2* PGVs, if they wish to do so. Yet there are clear disparities between which patients are evaluated for germline testing. From a narrative review of 20 articles related to *BRCA1/2* testing, Williams et al. concluded, “interventions are needed to reduce racial/ethnic disparities and improve assessment, provider recommendations, counseling, and testing among minority populations.”^6^

Measures to rectify the disparities between groups in who is evaluated for eligibility for testing are long overdue. To mitigate “inadequate access to health care services” among patients diagnosed with cancer, the recent Cancer Moonshot Initiative (version 2.0) recommended that payers eliminate requirements for pretest counseling by genetic specialists, training in genetics of other providers, and all patients with cancer be offered evaluation of testing.^7^ The Cancer Moonshot Initiative (version 2.0) also recommends that all patients diagnosed with cancer should be offered consultation with genetic specialists to ensure a truly informed clinician–patient discussion of the profound implications of genetic testing.^7^ Training more clinicians in the myriad complexities related to discussing genetic testing with their patients will be challenging. Still creating equity such that patients are afforded the opportunities that result from *BRCA1/2* testing must be considered.

Besides narrowing disparities between which groups of patients diagnosed with breast cancer are evaluated for testing, disparities between groups tested for *BRCA1/2* must be addressed as well. These disparities exist in part because the United States Preventive Services Task Force (USPSTF) and National Comprehensive Cancer Network (NCCN) Genetic/Familial High-Risk Assessment: Breast, Ovarian, and Pancreatic guidelines only endorse testing if the pretest probability that the patient carries a *BRCA1/2* PGV is “high,” and payers typically cover testing costs only if a patient meets guidelines’ criteria.^1,8,9^

Patients must pay out of pocket if they do not meet the NCCN and/or USPSTF testing criteria. This approach to testing might reasonably be considered economic discrimination—or health care inequity—against those who do not meet NCCN guidelines criteria and cannot afford to pay out of pocket.

Partly because of evidence that carrying a PGV appears to be similar among those who meet versus those not meeting NCCN and/or USPSTF guidelines testing criteria, the American Society of Breast Surgeons recommends that all patients with a diagnosis of breast cancer be offered testing.^10^ If adopted, testing would not be limited to those who meet guidelines criteria or can pay out of pocket.

One reason that other expert panels have not endorsed offering universal testing to all patients diagnosed with breast cancer is cost. The cost of testing for all the roughly 3.8 million current US breast cancer survivors, cascade testing of relatives, and then pursuing the recommended measures for prevention and early detection would be enormous. However, with the improved technology for genetic testing and the 2013 US Supreme Court decision related to gene patents, the cost of genetic testing has decreased in the last years, and, although there is considerable variability, the self-pay cost of testing has been reported to be as low as $250.^11–13^ After that decision, Mary-Claire King, who is credited with discovering *BRCA1/2* as well as much of the work characterizing *BRCA1/2* PGVs, lectured that “We should offer genetic testing to all women about the age of 30” and was quoted similarly in the ASCO Post in 2015.^14,15^ There are no guidelines that advocate such universal testing to adult women in the general population, but the lower costs have been a fundamental argument favoring offering testing to all patients diagnosed with breast cancer.

Another reason that expert panels have not endorsed universal genetic testing for patients diagnosed with breast cancer is the premise that genetic tests are unique as a category of lab tests that inform risk, in part due to the profound implications of test results for FMs.^16^ Although not studied extensively, barriers to sharing genetic testing information with FMs include concern for FM reaction, complexities of the information, lack of closeness, perceived relevance, and emotional impact.^17^

Despite the fact that false positive and false negative BRCA1/2 PGV results are rare and multi-gene panel testing is commonly used to screen for other breast cancer-predisposing PGVs, patients must be advised that not identifying a BRCA1/2 or other PGV does not mean that the patient’s risk for developing breast cancer is no different than the general population. Also, there are thought to be PGVs not yet identified that predispose to breast cancer.^17^

Perhaps the most common concern related to universal testing relates to the unacceptable “harm to benefit” ratio associated with a test that carries a significant likelihood of uncovering a germline variant—referred to as a “variant of uncertain significance” (VUS)—if all patients diagnosed with breast cancer—including those patients with lower pre-test probabilities—were given the option of being tested.^1,18^

Although there is some limited and mostly anecdotal evidence that VUSs have sometimes been inappropriately considered “actionable” by providers, the NCCN guidelines clearly state, and genetic test reports emphasis that practically no VUS should be considered “actionable.”^1^ Therefore, provided that genetic counselors or adhere to the NCCN guidelines, patients should be well informed of the significance of uncovering a VUS before testing is done and can judge for themselves, according to their own values and natures, whether the possibility of uncovering a VUS and other “harms” outweighs the benefits of proceeding with genetic testing.^1^

Ultimately, in weighing harms against benefits, germline testing is a very personal decision. In other words, one woman might see a 2% likelihood of carrying a *BRCA1/2* PGV as plenty of reason to want testing and, understandably, consider it a health inequity if she is denied coverage due to her inability to afford the out-of-pocket cost, while another woman might not be interested in testing, even if there is no associated out-of-pocket cost.

However, proponents of universal testing for patients diagnosed with breast cancer have argued that more patients could take advantage of measures proven to prevent cancers in *BRCA* PGV carriers and use drugs approved to treat *BRCA*-related cancers. Also, they suggest that the information provided by *BRCA* testing *per se* should be considered potentially valuable to patients, even if those results do not have “clinical utility” (i.e., are used to direct a change in treatment strategy), which is the typical criteria for including a test in the NCCN guidelines. Proponents suggest that patients “deserve” *BRCA* testing as a morally correct action.^19^ For example, the Secretary’s Advisory Committee on Genetics, Health, and Society reported “Clinical utility refers to…the value of information to the person being tested…Even if no interventions are available to treat or prevent disease, there may be benefits associated with knowledge of a result.”^20^

In summary, millions of patients will benefit from the opportunity—as advanced by Moonshot—to be evaluated and possibly tested for *BRCA1/2* PGVs. However, health equity will not occur unless the NCCN and/or USPSTF criteria for testing is expanded to include all those who wish to be tested, regardless of their pretest probability of carrying a *BRCA1/2* PGV and regardless of their ability to pay out of pocket. Patients should not continue to be denied health equity, as defined by the AMA, ACS, and ASCO, based on their inability to pay out of pocket for testing.

## Abbreviations Used

ACS: American Cancer Society
AMA: American Medical Association
ASCO: American Society of Clinical Oncology
ESMO: European Society of Medical Oncology
FM: Family Members
NCCN: National Comprehensive Cancer Network
PGV: Pathogenic Germline Variant
VUS: Variant of Uncertain Significance
USPSTF: United States Preventive Services Task Force

## References

[1] Daly MB, Pal T, Buys SS, et al. Genetic/Familial High-Risk Assessment: Breast, Ovarian, and Pancreatic, Version 2.2022, NCCN Clinical Practice Guidelines in Oncology March 9, 2022. Available from: https://www.nccn.org/guidelines/guidelines-detail?category=2&id=1503 10.6004/jnccn.2021.0001 33406487

[2] American Cancer Society. Advancing Health Equity—Addressing Cancer Disparities. Available from: https://www.cancer.org/about-us/what-we-do/health-equity.html

[3] Advancing Health Equity: A Guide to Language, narrative, and concepts. Available from: https://www.ama-assn.org/about/ama-center-health-equity/advancing-health-equity-guide-language-narrative-and-concepts

[4] Patel MI, Lopez AM, Blackstock W, et al. Cancer disparities and health equity: A policy statement from the American Society of Clinical Oncology. J Clin Oncol 2020;38(29):3439–3448. 32783672 10.1200/JCO.20.00642 PMC7527158

[5] Peters S. Striving for greater equality. A word from your president. Available from: https://www.esmo.org/about-esmo/president-s-letter/striving-for-greater-equality

[6] Williams CD, Bullard AJ, O’Leary M, et al. Racial/ethnic disparities in BRCA counseling and testing: A narrative review. J Racial Ethn Health Disparities 2019;6(3):570–583. 30963508 10.1007/s40615-018-00556-7

[7] President’s Cancer Panel. Closing Gaps in Cancer Screening: Connecting People, Communities, and Systems to Improve Equity and Access. 2022. Available from: https://prescancerpanel.cancer.gov/report/cancerscreening/

[8] Owens DK, Davidson KW, Krist AH, et al; US Preventive Services Task Force. Risk assessment, genetic counseling, and genetic testing for BRCA-related cancer: US Preventive Services Task Force Recommendation Statement. JAMA 2019;322(7):652–665. 31429903 10.1001/jama.2019.10987

[9] Aetna: Breast and ovarian cancer susceptibility gene testing, prophylactic mastectomy, and prophylactic oophorectomy. Available from: https://www.aetna.com/cpb/medical/data/200_299/0227.html

[10] Manahan ER, Kuerer HM, Sebastian M, et al. Consensus guidelines on genetic testing for hereditary breast cancer from the American Society of Breast Surgeons. Ann Surg Oncol 2019;26(10):3025–3031. 31342359 10.1245/s10434-019-07549-8 PMC6733830

[11] Cook-Deegan R, Niehaus A. After myriad: Genetic testing in the wake of recent supreme court decisions about gene patents. Curr Genet Med Rep 2014;2(4):223–241. 25401053 10.1007/s40142-014-0055-5 PMC4225052

[12] Beitsch PD, Whitworth PW, Hughes K, et al. Underdiagnosis of hereditary breast cancer: Are genetic testing guidelines a tool or an obstacle. J Clin Oncol 2019;37(6):453–460. 30526229 10.1200/JCO.18.01631 PMC6380523

[13] Insurance & Costs: Basser Center for BRCA. Penn Medicine. Available from: https://www.basser.org/genetic-testing-counseling-insurance-costs

[14] King MC. “Inherited breast and ovarian cancer and the resolution of paradox.” 2020. Available from: https://www.sabcsmeetingnews.org/mcguire-lecturer-echoes-call-for-universal-genetic-testing-for-brca1-2-mutations/

[15] Helwick C. Dr. Mary-Claire King proposes population screening in all young women for BRCA mutations. The ASCO Post. February 10, 2015. Available from: https://ascopost.com/issues/february-10-2015/dr-mary-claire-king-proposes-population-screening-in-all-young-women-for-brca-mutations/

[16] Green MJ, Botkin JR. “Genetic exceptionalism” in medicine: Clarifying the differences between genetic and non-genetic tests. Ann Intern Med 2003;138(7):571–575; doi: 10.7326/0003-4819-138-7-200304010-0003 12667027

[17] Dean M, Tezak AL, Johnson S, et al. Sharing genetic test results with family members of BRCA, PALB2, CHEK2, and ATM carriers. Patient Educ Couns 2021;104(4):720–725. 33455826 10.1016/j.pec.2020.12.019 PMC8005459

[18] Burke W, Parens E, Chung WK, et al. The challenge of genetic variants of uncertain clinical significance: A narrative review. Ann Intern Med 2022;175(7):994–1000; doi: 10.7326/M21-4109 35436152 PMC10555957

[19] Subbiah V, Kurzrock R. Universal germline and tumor genomic testing needed to win the war against cancer: Genomics is the diagnosis. J Clin Oncol 2023;41(17):3100–3103. 36930859 10.1200/JCO.22.02833 PMC10256401

[B20] 20. US Department of Health and Human Services. Secretary’s Advisory Committee on Genetics, Health, and Society-Coverage and Reimbursement of Genenetic Tests and Services. 2006. Available from: https://osp.od.gov/wp-content/uploads/NIH_Guidelines.pdf

